# Inhibition of Tumor Growth via Systemic siRNA Delivery Using Reducible Bile Acid-Conjugated Polyethylenimine

**DOI:** 10.3390/polym10090953

**Published:** 2018-08-27

**Authors:** Yue Yin, Jung Eun Lee, Nak Won Kim, Jong Han Lee, Su Yeon Lim, E Seul Kim, Ji Won Park, Min Sang Lee, Ji Hoon Jeong

**Affiliations:** School of Pharmacy, Sungkyunkwan University, Suwon 16419, Korea; yinyue2013@skku.edu (Y.Y.); jelee1213@naver.com (J.E.L.); paradiseboom@naver.com (N.W.K.); chq29kr@gmail.com (J.H.L.); suuyeon37@skku.edu (S.Y.L.); seul4146@naver.com (E.S.K.); jwjwpar@gmail.com (J.W.P.)

**Keywords:** small interfering RNA, deoxycholic acid, stabilized polyplex, disulfide crosslinking, systemic gene therapy

## Abstract

RNA interference (RNAi), mediated by small interfering RNA (siRNA), has been considered as a potential therapeutic agent for cancer owing to its ability to suppress target genes in a sequence-specific manner. In this study, a conjugate of the low molecular weight (M_W_) polyethylenimine (PEI) (M_W_ 1800) and deoxycholic acid (DA) was further modified with 4-fluorothiophenol (FTP) (TP-DA-PEI) to achieve systemic siRNA delivery. The thiophenol group would be involved with disulfide bonds between the polymer chains and siRNA modified with free thiols (thiol-siRNA) to form and π–π interactions between the pendent aromatic groups and coprostane ring of the bile acid. The TP-DA-PEI conjugates could generate stable nanoparticles with thiol-siRNA. The TP-DA-PEI conjugate not only achieved enhanced intracellular uptake, serum stability, and transfection efficiency, but also showed high accumulation of TP-DA-PEI/thiol-siRNA polyplexes and significant tumor growth inhibition effect in tumor-bearing mice after systemic administration.

## 1. Introduction

Small interfering RNA (siRNA), double-stranded RNA with 19–23 base pairs, can effectively downregulate the targeted gene expression. siRNA has been used as a potential drug for cancer therapy based on the modulation of cell cycle and proliferation, induction of apoptosis, down-regulation of oncogenes, and suppression of angiogenesis [1]. Systemic delivery of siRNA provides therapeutic advantages over local delivery, especially in the treatment of cancer that could be localized in the deep tissues or disseminated throughout the body, that is, metastatic cancers [2,3,4]. However, the inherent susceptibility of siRNA to degradation by nucleases in the blood and its poor cellular uptake have been considered major challenges for the clinical use of the systemic siRNA therapy [5,6,7].

Polymer- or lipid-based non-viral gene carriers have long been developed as alternatives for virus-driven carriers, which still have safety concerns [8,9,10]. Most non-viral carriers form nanoparticles with siRNA based on electrostatic interactions. Because of the short length and rigid double-stranded structure of siRNA, however, many conventional polymeric carriers designed for nucleic acid drugs with relatively long and flexible structures, such as plasmid DNA, turned out to be unsuitable for the systemic delivery of siRNA because they form less compact complexes with siRNA than with plasmid DNA [11,12]. In our previous study, we showed that non-toxic low molecular weight polyethylenimine (PEI, 1.8 kDa) conjugated with an amphiphilic bile acid can form a stable complex with siRNA and mediate efficient cellular transfer of siRNA [13,14]. Among the conjugates of various bile acids, a deoxycholic acid-PEI conjugate (DA-PEI) exhibited superior ability in cellular gene transfer and was employed as a carrier for in vitro and in vivo gene delivery [15,16,17,18].

In the present study, the DA-PEI was modified with a thiophenolic derivative to generate more stable polyplex with thiol-modified siRNA (TP-DA-PEI/thiol-siRNA). A derivative of fluorobenzene, 4-fluorothiophenol (FTP), was used as a pendent group for the formation of disulfide bonds between polymer chains and with thiol-siRNAs. The disulfide bonds would provide additional stability to the polyplex in the blood during systemic circulation, but readily cleaved in a reductive intracellular environment after cellular uptake [19,20]. Furthermore, the π–π interactions between the aromatic pendent groups of the polymer chains and between the pendent group and coprostane ring of the bile acid may also contribute in the formation of robust polyplex that could maintain its colloidal stability in the blood stream after systemic administration. Cellular and systemic delivery of vascular endothelial growth factor (VEGF)-targeting siRNA using TP-DA-PEI were observed and the anticancer effect of the systemically administered siRNA was assessed in an animal tumor model.

## 2. Materials and Methods

### 2.1. Materials

Low molecular weight branched polyethylenimine (bPEI, M_W_ 1800), high molecular weight branched polyethylenimine (bPEI, M_W_ 25,000), deoxycholic acid (DA), dicyclohexyl carbodiimide (DCC), *N*-hydroxysuccinimide (NHS), 4-Fluorothiophenol, heparin sodium salt, tris-acetate-ethylenediaminetetraacetic acid (TAE) buffer, dithiothreitol (DTT), 3-(4,5-dimethyl-2-thiazolyl)-2,5-diphenyl-2H-tetrazolium bromide (MTT), phosphate buffered saline (PBS), and 4-(2-hydroxyethyl)-1-piperazineethanesulfonic acid (HEPES) were purchased from Sigma-Aldrich (St. Louis, MO, USA). For cell culture products, fetal bovine serum (FBS) and Roswell Park Memorial Institute 1640 medium (RPMI 1640) were obtained from Invitrogen (Carlsbad, CA, USA). The RNA Purification kit was purchased from Qiagen (Hilden, Germany). Iscript cDNA synthesis and quantitative reverse transcription polymerase chain reaction (RT-qPCR) kits were purchased from Bio-Rad (Berkeley, CA, USA). The ELISA kit was purchased from R&D systems (Minneapolis, MN, USA). All chemicals and reagents were of analytical grade and used as received unless otherwise mentioned.

### 2.2. Small Interfering RNAs (siRNAs) Design

All types of siRNA and thiol-siRNA were supplied by Bioneer (Daejeon, South Korea). The sequences of the VEGF siRNA and thiol-siRNA were 5′-GGAGUACCCUGAUGAGAUCdTdT-3′ (sense) and 5′-GAUCUCAUCAGGGUACUCCdTdT-3′ (antisense). The sequences of scrambled siRNA and thiol-siRNA were 5′-ACGCGUAACGCGGGAAUUUdTdT-3′ (sense) and 5′-AAAUUCCCGCGUUACGCGUdTdT-3′ (antisense). The sequences of the green fluorescence protein (GFP) siRNA and thiol-siRNA were 5′-AACUUCAGGGUCAGCUUGC-3′ (sense) and 5′-GCAAGCUGACCCUGAAG UU-3′ (antisense).

### 2.3. Synthesis of DA-PEI and TP-DA-PEI Conjugates 

The PEI (M_W_ 1800) conjugated with DA (DA-PEI) was synthesized as described in previous studies [15]. Briefly, 1 g of DA (2.5 mmol) dissolved in dichloromethane was activated by dicyclohexylcarbodiimide (DCC, 1.57 g, 7.6 mmol) and *N*-hydroxysuccinimide (NHS, 0.88 g, 7.6 mmol) (DA/DCC/NHS stoichiometric molar ratio = 1:3:3). The activated DA was precipitated in hexane and dried under vacuum. This conjugate was synthesized by slowly adding the activated DA to PEI in dichloromethane. The molar feed ratio for DA to PEI was 3:1. The product was dried using a rotary evaporator, dissolved in 0.1 M hydrochloric acid (HCl), and precipitated in cold acetone. The precipitate was dried in air condition. The resultant product was then dissolved in deionized water, filtered, and lyophilized. To generate different degree substitutions of 4-Fluorothiophenol (FTP) conjugates, 0.3 g of DA-PEI dissolved in dimethyl sulfoxide (DMSO) (Sigma-Aldrich, St. Louis, MO, USA) was mixed with different desired amounts of FTP dissolved in DMSO, respectively. The molar feed ratios for DA-PEI to FTP were 1:1, 1:3, and 1:5, respectively. After reaction, the final products TP(1)-DA-PEI, TP(3)-DA-PEI, and TP(5)-DA-PEI were purified by dialysis and lyophilized. The degree of substitution of FTP was determined by using ^1^H NMR.

### 2.4. Preparation of TP-DA-PEI/thiol-siRNA

To prepare various polyplexes, the conjugates synthesized above were performed at desired weight ratios with siRNA and thiol-siRNA, respectively. In the case of bPEI25k/siRNA and DA-PEI/siRNA groups, a fixed amount of siRNA (0.5 μg) was mixed with the desired amount of conjugates in 50 μL of PBS. In terms of the disulfide crosslinking, 0.5 μg of siRNA or thiol-siRNA was mixed with the desired amount of conjugates (TP(1)-DA-PEI, TP(3)-DA-PEI, and TP(5)-DA-PEI) in 50 μL of 10 mM HEPES buffer (pH 8.0), respectively. Then, all polyplexes were incubated for 6 h by exposure to oxygen condition at room temperature.

The binding ability of siRNA and thiol-siRNA with polymeric carriers was examined by gel electrophoresis. The siRNA and thiol-siRNA (1.0 μg) were respectively complexed with various conjugates (DA-PEI, TP(1)-DA-PEI, TP(3)-DA-PEI, and TP(5)-DA-PEI) at polymer/siRNA or thiol-siRNA weight ratios ranging from 0 to 2, and then each kind of polyplex was incubated for the desired time. Sequentially, polymer/siRNA or thiol-siRNA polyplexes (20 μL) were loaded on 1% (*w*/*v*) agarose gel containing ethidium bromide (Sigma-Aldrich, St. Louis, MO, USA) in 1X TAE buffer (10 mM Tris/HCl, 1% (*v*/*v*) acetic acid, 1 mM EDTA) with 4 μL of 6× loading dye (Sigma-Aldrich, St. Louis, MO, USA). These gels were allowed to run at 120 V for 20 min in 1X TAE buffer. The mobility of siRNA or thiol-siRNA was visualized with UV light using a Dual UV Transilluminator (Core Bio System, Huntington Beach, CA, USA).

In order to confirm the formation of disulfide bonds, each polyplex containing 1 μg of siRNA or thiol-siRNA at polymer/siRNA weight ratio of 3:1 was adopted with 100 μg of heparin and 1 μL of 1 M DTT solution. The sample solution containing these polyplexes was loaded on 1% agarose gel containing ethidium bromide and electrophoresed at 120 V for 20 min in 1X TAE buffer and these gels were also visualized by exposure to the Dual UV Transilluminator. Furthermore, the size and surface charge of several polyplex nanoparticles were measured by a dynamic light scattering device (Zetasizer Nano, Malvern, Worcestershire, UK).

### 2.5. Serum Stability

To confirm serum stability, siRNA or thiol-siRNA was complexed with bPEI25k at polymer/siRNA or thiol-siRNA weight ratio of 1:1, and with DA-PEI and TP(3)-DA-PEI at polymer/siRNA or thiol-siRNA weight ratio of 3:1, respectively. Polyplexes were incubated for 6 h by exposing to oxygen condition at room temperature. The prepared polyplexes were incubated in RPMI1640 medium (Invitrogen, Carlsbad, CA, USA) containing 50% FBS at 37 °C for predetermined periods of time (0, 1, 2, 4, 6, 12, 24, 36, and 48 h). To fully release the siRNA or thiol-siRNA condensed with different polymers, 100 μg of heparin and 1 μL of 1M DTT were added to each sample solution containing 1 μg of siRNA or thiol-siRNA. The stability of the siRNA or thiol-siRNA was examined by electrophoresis in a 1% (*w*/*v*) agarose gel containing ethidium bromide and all samples were mixed with loading dye. Electrophoresis was conducted at 120 V for 20 min in 1X TAE buffer. After electrophoresis, the gels were visualized using the UV Transilluminator.

### 2.6. In Vitro Gene Silencing Test of TP-DA-PEI/thiol-siRNA 

To compare the gene silencing efficiency in the presence and absence of serum, TP(3)-DA-PEI was selected as the optimal conjugate for this stage. With the procedure described above, A549 cells were seeded at 12-well culture plates at a density of 1.0 × 10^5^ cells per well in 1.0 mL of culture medium and then incubated for 24 h before transfection. Cells were divided into two groups, then the medium without serum and with 10% serum were changed during a 4 h transfection period. These prepared polyplexes containing 0.5 μg of siRNA or thiol-siRNA complexed with DA-PEI and TP(3)-DA-PEI at polymer/siRNA weight ratio of 3:1, respectively, were transfected into cells. The bPEI25k/siRNA and bPEI25k/thiol-siRNA at polymer/siRNA weight ratio of 1:1 were used as controls. After 4 h incubation, the transfection medium was removed and supplemented with RPMI1640 medium containing fresh serum and the cells were continuously incubated for 48 h at 37 °C. To measure the extent of GFP expression, the transfected cells were washed with PBS and treated with 200 μL of 1X cell lysis buffer (Promega, Madison, WI, USA) for each well, which was followed by centrifugation using a centrifuge (Hanil Science Industrial Co., Ltd., Incheon, South Korea) at 14,000 rpm for 10 min. After centrifugation, the cell supernatant was obtained for quantitative analysis by GloMax 20/20 luminometer (Promega, Madison, WI, USA). The relative GFP expression level was calculated based on the GFP expression percentage of non-treated A549 cell used as a 100% control. 

To determine VEGF gene silencing effect of polyplexes, thiol-VEGF siRNAs were complexed with DA-PEI and TP(3)-DA-PEI at weight ratio of 3:1. The TP(3)-DA-PEI/thiol-scrambled siRNA and bPEI25k/thiol-VEGF siRNA were also used as controls. The prepared polyplexes were treated to A549 cells in the RPMI1640 medium containing 10% FBS. After 4 h incubation, the medium was replaced with fresh culture medium containing 10% FBS, and the cells were further incubated for 24 h. The medium samples were collected from cell culture supernatants and the particulates in the medium were immediately removed by centrifugation. The amount of VEGF protein was determined using Quantikine^®^ VEGF ELISA kit (R&D Systems, Minneapolis, MN, USA) according to the manufacturer’s instructions. The absorbance was measured on a microplate reader (Bio-Tek Instrument, Winooski, VT, USA) at 450 nm. The cellular level of VEGF mRNA was analyzed by a reverse transcriptase polymerase chain reaction (RT-PCR). The A549 cells were transfected with various polyplexes. After 18 h incubation, total RNA was separately extracted from the cells using RNeasy Mini Kit (QIAGEN, Seoul, Korea) according to the manufacturer’s instructions. RT-PCR was conducted with 0.5 μg of total RNA by SuperScript™ Ш One-step RT-PCR system (Bio-Rad, Hercules, CA, USA) with Platinum Taq DNA polymerase (Thermo Fisher Scientific, Waltham, MA, USA). The cDNA synthesis was performed at the following thermal cycling condition at 25 °C for 5 min, at 42 °C for 30 min, and at 85 °C for 5 min. The primer sequences for VEGF were sense: 5′-GGGCAGAATCATCACGAAGT-3′ and antisense: 5′-AAATGCTTTCTCCGCTCTGA-3′ (359 bp), and the primer sequences for β-actin were sense: 5′-GTGCGTGACATTAAGGAG-3′ and antisense: 5′-CTAAGTCATAGTCCGCCT-3′ (520 bp). The samples were amplified through incubation at 94°C for 5 min, 30 cycles of denaturation at 94 °C for 45 s, annealing at 54 °C for 45 s and extension at 72 °C for 1 min, and final incubation at 72 °C for 7 min. The PCR products were analyzed on 1% (*w*/*v*) agarose gel containing ethidium bromide.

### 2.7. Cytotoxicity Assay

The relative cytotoxicity of polyplexes was investigated by MTT assay. A549 cells were seeded at a density of 1.0 × 10^4^ cells per well in 96-well plates. After incubation for 24 h, various polyplexes containing siRNA or thiol-siRNA (0.05 μg siRNA per well), complexed with DA-PEI or TP(3)-DA-PEI at polymer/siRNA weight ratio of 3:1, were added in each culture well. After 4 h transfection, the cells were continuously incubated in a fresh serum-containing medium for 24 h at 37 °C. Subsequently, 20 μL of MTT solution was added into each culture well. After 4 h incubation, all medium of each culture well was replaced by 200 μL of DMSO until all insoluble formazan crystals were fully dissolved. Absorbance was measured at 490 nm in a microplate reader (Bio-Tek Instrument, Winooski, VT, USA). The cell viability was determined relative to the untreated control cells.

### 2.8. Intracellular Uptake of TP-DA-PEI/thiol-siRNA

For the intracellular uptake study, A549 cells were grown in confocal imaging dishes (35 mm Glass Bottom Dish, SPL Life Sciences, Pocheon, South Korea) for confocal microscopy. The Cy3-labeled siRNA and Cy3-labeled thiol-siRNA were complexed with DA-PEI and TP(3)-DA-PEI at polymer/siRNA weight ratio of 3:1, respectively. The bPEI25k/thiol-siRNA polyplex at weight ratio of 1:1 was used as control. The A549 cells were transfected with these different polyplexes for 4 h, and then washed three times with PBS. The nucleoli were stained with 4′,6-diamidino-2-phenylindole (DAPI, Invitrogen, Seoul, South Korea). The cells were then fixed by submerging the cells in 10% buffered formaldehyde (Hedwin Corp., Baltimore, MD, USA). The subcellular localization of the polyplexes was visualized by a Zeiss LSM 510 laser scanning confocal fluorescence microscope (Carl Zeiss MicroImaging GmbH, München, Germany).

To quantitatively evaluate the cellular uptake, fluorescence activated cells sorting (FACS) were also analyzed using Cy3-labeled siRNA and Cy3-labeled thiol-siRNA. A549 cells were seeded in 12-well plates at a density of 1.0 × 10^5^ cells per well. After 24 h incubation, cells were transfected with the different polyplexes containing bPEI25k, DA-PEI, and TP(3)-DA-PEI for 4 h at 37 °C. After washing three times with PBS, the transfected cells were harvested by trypsin digestion, and then washed three times with PBS again. The extent of Cy3-labeled siRNA or Cy3-labeled thiol-siRNA was immediately monitored by a flow cytometer (Guava easyCyte™ Flow Cytometer, Merck Millipore, Billerica, MA, USA) at excitation of 550 nm and emission of 570 nm. Data were further processed using InCyte software (Guava easyCyte™ Flow Cytometer, Merck Millipore, Billerica, MA, USA). 

### 2.9. In Vivo Imaging

All animal experiments were performed in accordance with the guidelines provided by the Institutional Animal Care and Use Committee at Sungkyunkwan University (SKKUIACUC2018-05-09-1). A549 cells (5 × 10^6^ for each mouse) were injected in the flank region of female nude mice (nu/nu) at six weeks of age. When the tumor volume reached approximately 200 mm^3^, various polyplexes containing bPEI25k/Cy5.5-labeled thiol-siRNA, DA-PEI/Cy5.5-labeled thiol-siRNA, and TP(3)-DA-PEI/Cy5.5-labeled thiol-siRNA at siRNA dose of 1 mg/kg were intravenously injected into the mice via the tail vein. PBS solution was also administrated as a control. Images were performed by an in vivo optical analysis system equipped with Analysis Workstation software (Optix^®^ MX3, Advanced Research Technologies, Montreal, QC, Canada) for quantitative analysis at the excitation wavelength of 678 nm and the emission wavelength of 694 nm at 12 h post injection. Mice were anesthetized with enflurane and maintained throughout the imaging process. The near-infrared fluorescence images were acquired with a 60 s exposure time. At the same time point, mice were sacrificed, then tumors and normal tissues (liver, lung, spleen, and kidney) were dissected, followed by washing the surface with PBS for the ex vivo fluorescence imaging.

### 2.10. Tumor Growth Inhibition

To investigate the in vivo anticancer effect, A549 cells (5 × 10^6^ for each mouse) were injected subcutaneously in the flank region of female nude mice (nu/nu) aged six weeks old. When the tumor volume reached approximately 100 mm^3^, the mice were randomly divided into four groups (*n* = 6 for each group) and intravenously administrated with three types of polyplexes: bPEI25k, DA-PEI, and TP(3)-DA-PEI complexed with thiol-VEGF siRNA (1 mg/kg), respectively. The control group was intravenously injected with PBS solution. In all cases, the intravenous administrations were continued five times at 0, 3, 6, 10, and 15 day intervals. Each mouse was earmarked and followed individually throughout the experiments. Tumor growth was measured by measuring the length and width of each tumor with a caliper daily over 50 days until the mice were euthanized. The estimated volume was calculated according to the following formula: tumor volume (mm^3^) = length × width^2^/2.

## 3. Results and Discussion

### 3.1. Formation and Characterization of TP-DA-PEI/thiol-siRNA

**Scheme 1:**
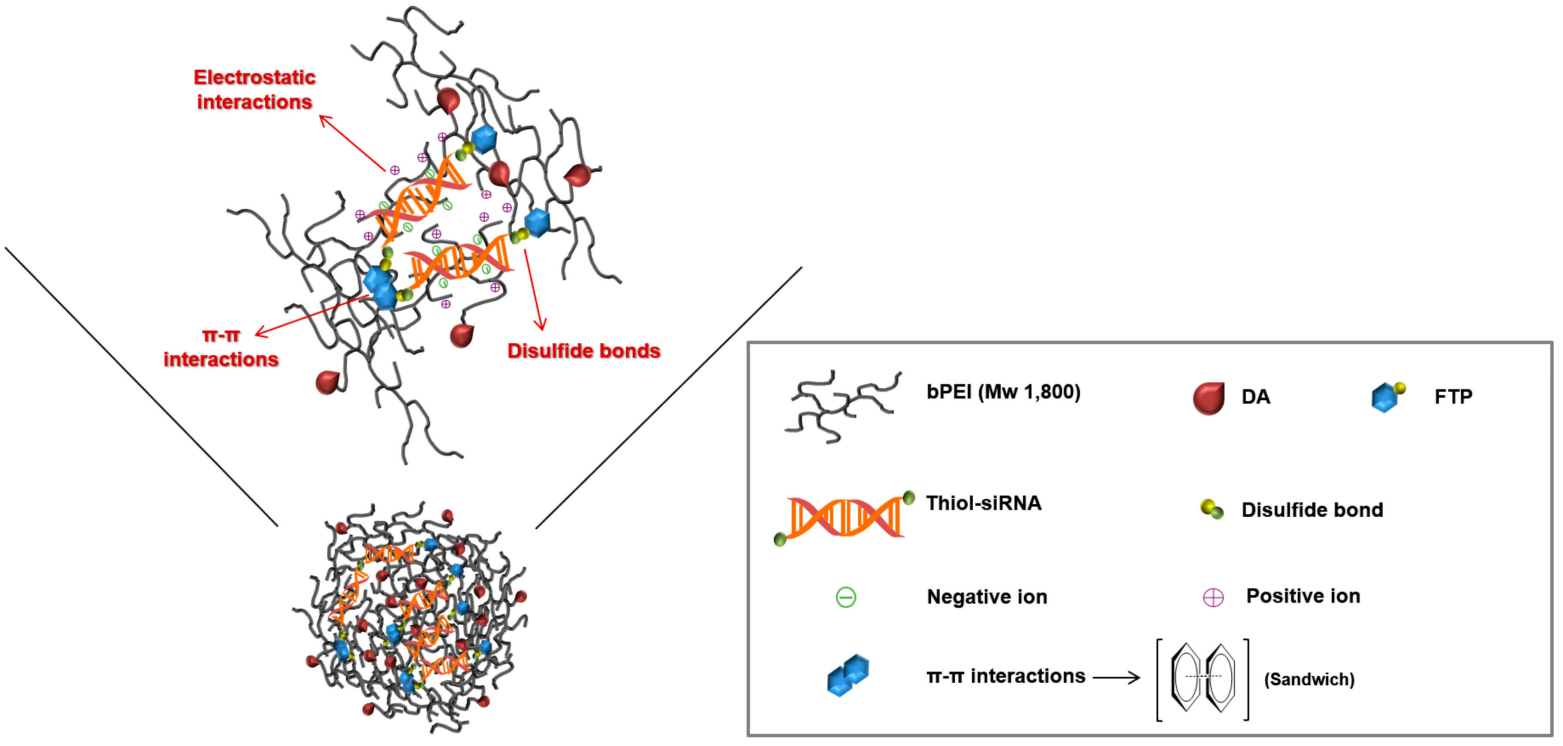
Overview of 4-fluorothiophenol (FTP)-deoxycholic acid (DA)-polyethylenimine (PEI) conjugates/thiol-modified small interfering RNA (siRNA) polyplex forming process including the amphiphilic accumulation, electrostatic interaction, the formation of disulfide bonds and π–π stacking forces. M_W_—molecular weight.

We previously showed that low molecular weight PEI conjugated with amphiphilic deoxycholic acid (DA-PEI) could form stable polyplex nanoparticles with siRNA and mediate efficient cellular delivery of siRNA without significant cytotoxicity [15,16,17,18]. The DA-PEI also successfully suppressed tumor growth by delivering therapeutic siRNA to the tumor tissue after the direct injection of the polyplex into the solid tumor [21]. To design a siRNA carrier with improved stability for systemic siRNA delivery, DA-PEI was further modified by introducing thiophenonyl groups (TP-DA-PEI). When complexed with siRNA having thiol groups at each end, the TP-DA-PEI conjugate formed a stable nano-sized polyplex. In addition to electrostatic interactions, covalent disulfide linkages between the aromatic pendent group and thiol-siRNA and π–π stacking forces between the aromatic groups and coprostane ring of deoxycholic acid may contribute to form a strongly entangled structure inside the polyplex, maintaining structural stability in the blood circulation after systemic administration (Scheme 1).

**Figure 1:**
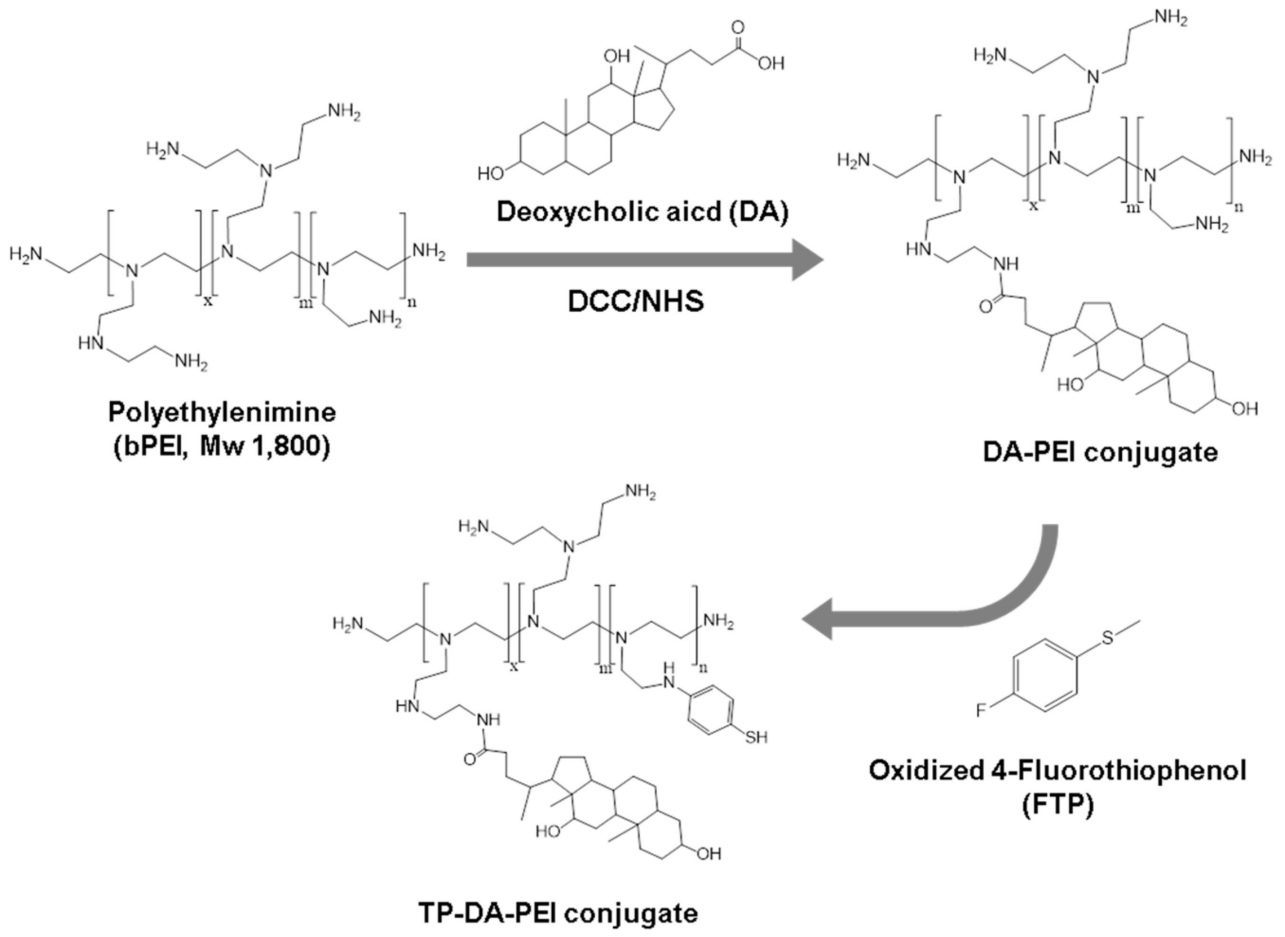
Synthesis of the TP-DA-PEI conjugates. M_W_—molecular weight.

DA-PEI conjugate was synthesized using DCC/NHS chemistry to form an amide bond between the carboxylic acid of DA and the primary amine group of low molecular weight PEI (PEI, M_W_ 1800), as previously described [14]. The degree of substitution of DA/PEI was 2.5, as determined by ^1^H NMR. TP-DA-PEI conjugate was synthesized by a nucleophilic replacement of the fluorine of FTP with a primary amine of DA-PEI (Figure 1) at different reaction ratios (stoichiometry molar feed ratio of DA-PEI to FTP = 1:1, 1:3, and 1:5) (Table 1). The synthesis of the TP-DA-PEI conjugates was confirmed using ^1^H NMR (Figure S1). The emergence of an aromatic ring proton signal (–C_6_H_5_, 6.50–7.50 ppm) indicated that FTP was successfully conjugated to PEI (M_W_ 1800). The degree of substitution was calculated by the ratio between the integral values of the aromatic ring protons (–C_6_H_5_, 6.50–7.50 ppm) and original alkyl group protons of DA (–CH_3_, 0.90 ppm). The ratio of the integrals of the signals corresponding to the protons was in good agreement with the presumptive structure of the polymers.

**Figure 2:**
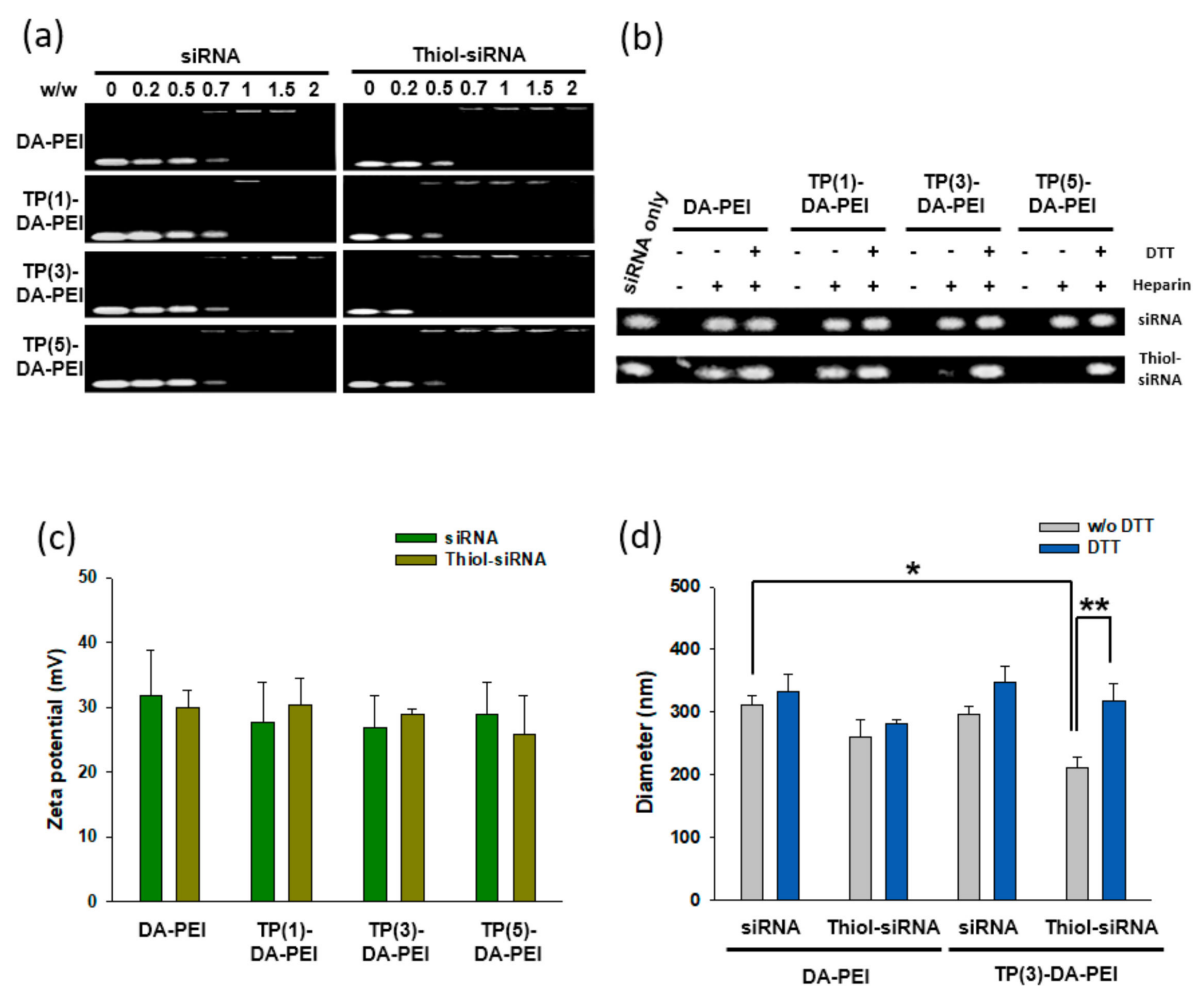
Gel retardation assay of (**a**) small interfering RNA (siRNA) condensed with DA-PEI, TP(1)-DA-PEI, TP(3)-DA-PEI, and TP(5)-DA-PEI at polymer/siRNA weight ratios from 0 to 2 and (**b**) the examination of the formation of disulfide bonds in various polyplexes at polymer/siRNA weight ratio of 3 on 1% (W/V) agarose gel and stained with ethidium bromide. (**c**) The zeta potential values of TP-DA-PEI at polymer/siRNA weight ratio of 3. The results are presented as means ± SD. (**d**) Effect of the formation of disulfide bonds on the sizes of polyplexes (polymer/siRNA weight ratio = 3), measured by dynamic light scattering (DLS) analysis. The results are presented as means ± SD and analyzed using Student’s *t*-test (*n* = 3, * *p* < 0.05, ** *p* < 0.01).

To observe the formation of polyplex of TP-DA-PEI conjugates with either siRNA or thiol-siRNA, the band mobility shift in an electrophoretic field was observed (Figure 2a). The TP-DA-PEI conjugate can successfully form a polyplex with both siRNA and thiol-siRNA at the weight ratio of above 1 (polymer/siRNA, *w*/*w*), while thiol-siRNA completely condensed them at the weight ratio of above 0.7. This may be a result of the additional contribution of disulfide bonds and π–π interactions to the formation of the compact polyplex structure [22]. The formation of a disulfide bond between TP-DA-PEI and thiol-siRNA was further confirmed by a heparin competition assay and DTT treatment (Figure 2b). While unmodified siRNA was readily released from the polyplex by adding heparin regardless of DTT treatment, thiol-siRNA complexed with TP(3)-DA-PEI and TP(5)-DA-PEI was not released in the presence of heparin. When treated with DTT before adding heparin, thiol-siRNA was then released from polyplex. The result suggests that the retardation of siRNA band mobility was a result of the covalent attachment of thiol-siRNA to the polymers via reducible disulfide linkages.

All the formulations of TP-DA-PEI showed similar zeta-potential values, suggesting that the addition of thiophenol moiety to DA-PEI did not significantly influence the surface charge of polyplexes (Figure 2c). The formation of disulfide bonds between TP(3)-DA-PEI and thiol-siRNA resulted in smaller particle size, compared with other formulations. The particle size of TP(3)-DA-PEI/thiol-siRNA was 210 ± 25 nm in diameter, which became larger (300 ± 20 nm) by the reduction of disulfide bonds in the presence of DTT. This suggests the additional contribution of disulfide bonds to making the polyplex more compact and stable.

### 3.2. In Vitro Gene Silencing Efficiency of TP-DA-PEI/thiol-siRNA

**Figure 3:**
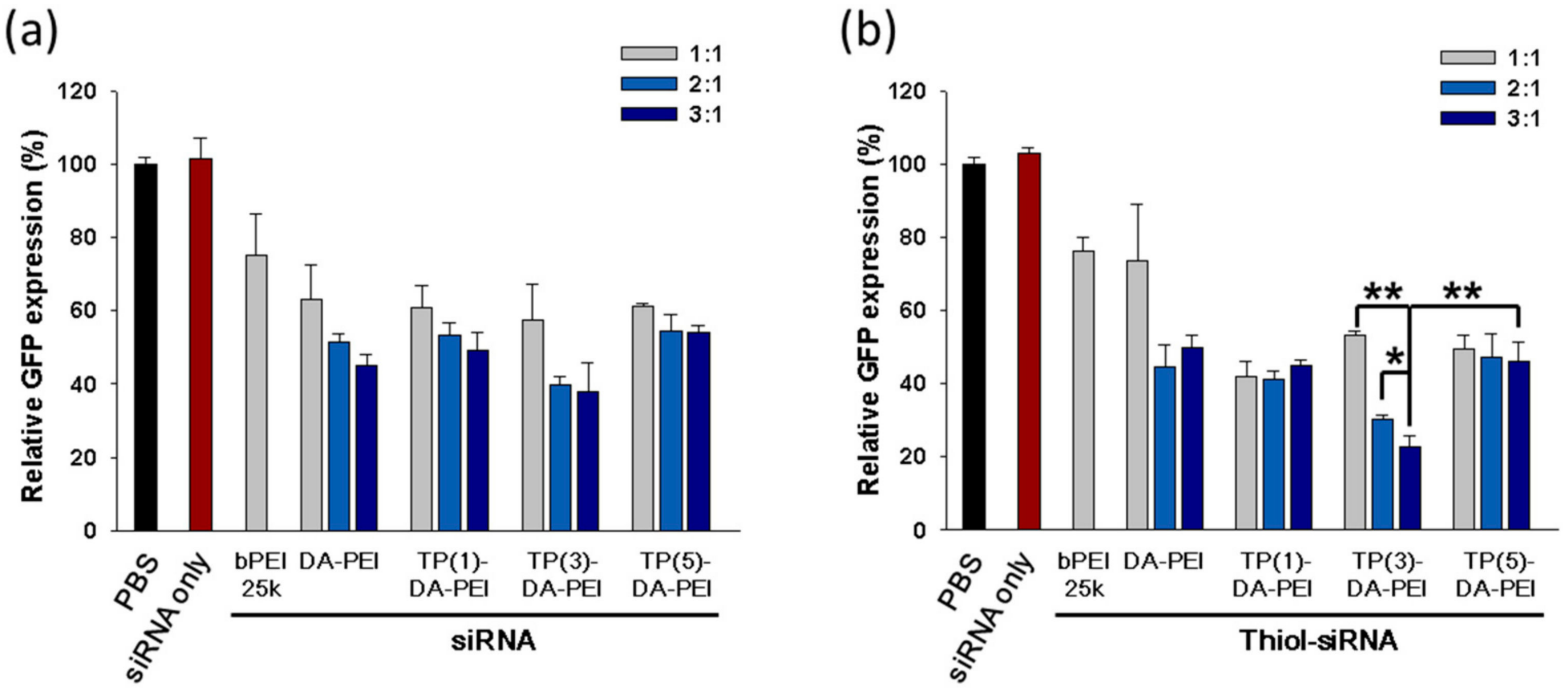
Green fluorescence protein (GFP) gene silencing efficiency of bPEI25k, DA-PEI, TP(1)-DA-PEI, TP(3)-DA-PEI, and TP(5)-DA-PEI condensed with (**a**) GFP siRNA and (**b**) thiol-GFP siRNA, respectively, at various polymer/siRNA weight ratios of 1, 2, and 3 in GFP overexpressing A549 cells. The results are presented as means ± SD and analyzed using Student’s *t*-test (*n* = 3, * *p* < 0.05, ** *p* < 0.01).

The transfection ability of TP-DA-PEI conjugates was investigated in A549 cells overexpressing GFP using siRNA or thiol-siRNA targeting GFP. Both TP-DA-PEI/siRNA and TP-DA-PEI/thiol-siRNA polyplexes efficiently suppressed the GFP expression as the polymer/siRNA weight ratio increased (Figure 3a,b). In particular, TP(3)-DA-PEI/thiol-siRNA at weight ratio of 3 achieved the strongest GFP silencing effect, compared with other groups. However, polyplexes assembled with TP(5)-DA-PEI and TP(3)-DA-PEI showed similar target gene silencing efficiency to DA-PEI. This result suggests that the balance of stabilizing forces, including disulfide bonds and π–π interactions, may play an important role in cellular gene delivery and unpacking of polyplex in the cytosolic environment. The reduction-sensitive disulfide bonds of the polyplex are expected to remain stable in the extracellular environment and regularly detach under intracellular reducing conditions via disulfide cleavage [11]. Oxidation-based crosslinking between thiolated polymers resulted in enhanced gene transfection because disulfide bonds (bond energy: ~60 kcal mol^−1^) in the polycations were hydrolytically stable and could be cleaved in the reductive cytosolic conditions, because of a high concentration (1–10 mM) of reduced glutathione (GSH) in the cytosol [23]. In addition, the GSH concentration of tumor cells was reported to be approximately seven-fold higher than that of normal cells [24,25]. On the basis of these results, TP(3)-DA-PEI/thiol-siRNA at weight ratio of 3 was used for further experiments.

### 3.3. Serum Stability and In Vitro Gene Silencing of TP-DA-PEI/thiol-siRNA

**Figure 4:**
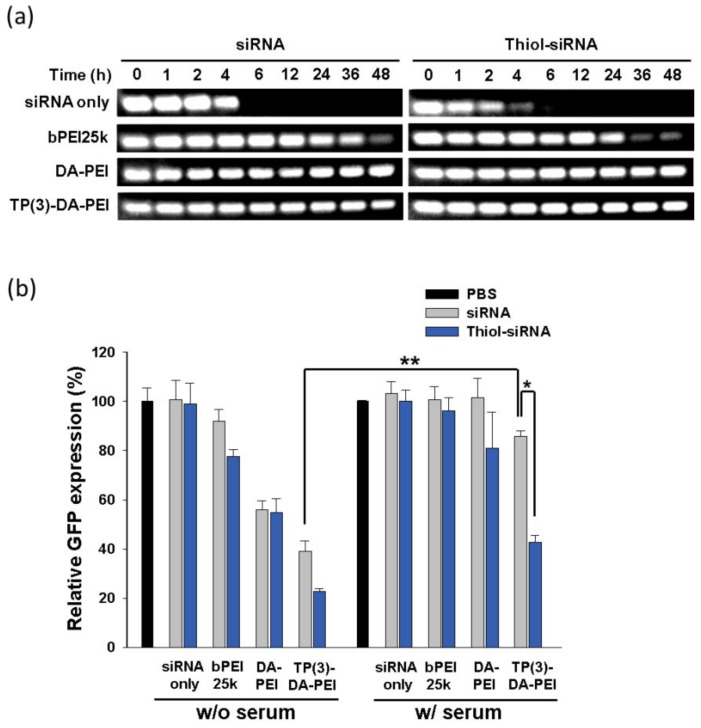
(**a**) Stability of siRNA and thiol-siRNA in the presence of 50% serum. Either siRNA or thiol-siRNA was complexed with the indicated polymers: bPEI25k (polymer/siRNA weight ratio = 1), DA-PEI (weight ratio = 3) and TP(3)-DA-PEI (weight ratio = 3). (**b**) Comparison of GFP gene silencing capability of various formulations in presence of 50% serum. The results are presented as means ± SD and analyzed using Student’s *t*-test (*n* = 3, * *p* < 0.05, ** *p* < 0.01).

To evaluate the stability of siRNA or thiol-siRNA under serum condition, various polyplexes were incubated in the presence of 50% fresh FBS. While naked siRNA and thiol-siRNA were almost completely degraded within 6 h, the polyplexes formed from DA-PEI and TP(3)-DA-PEI efficiently protect the siRNA from degradation up to 48 h in the presence of 50% FBS (Figure 4a). The presence of serum also significantly affected transfection efficiencies of the polymeric carriers (Figure 4b). Among the formulations using bPEI25k, DA-PEI, and TP(3)-DA-PEI, TP(3)-DA-PEI/thiol-siRNA exhibited a meaningful level of suppression of the target gene expression in the presence of serum, indicating that the disulfide bonds in the polyplex would significantly contribute to the serum stability. The result also suggests that TP(3)-DA-PEI/thiol-siRNA would protect siRNA from enzymatic digestion and maintain polyplex stability in the blood stream.

**Figure 5:**
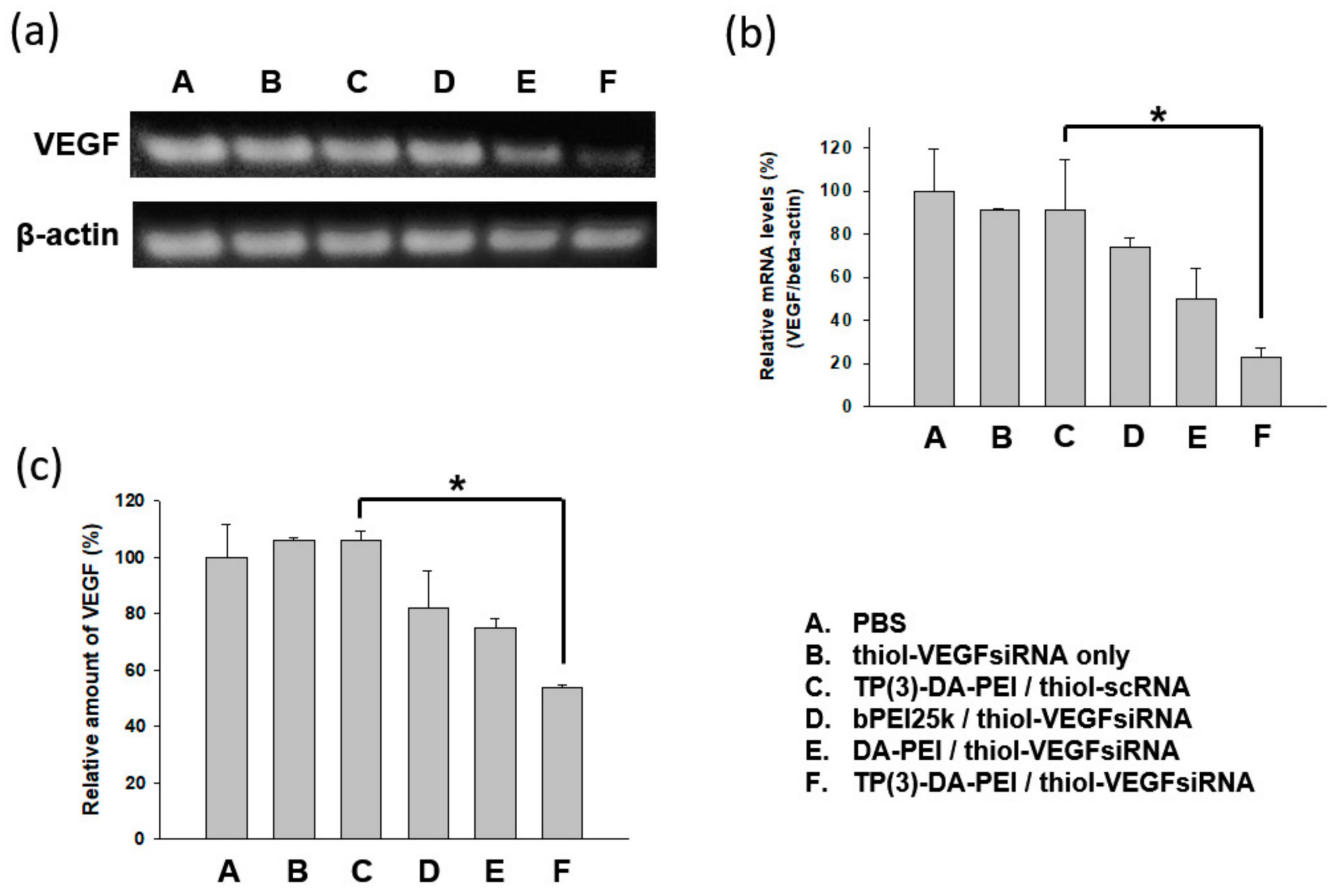
Vascular endothelial growth factor (VEGF) mRNA expression in A549 cells investigated by reverse transcription polymerase chain reaction (RT-PCR) showing in (**a**) the representative image (**b**) the statistical bar graph of VEGF/β-actin relative ratios (compared with control group). (**c**) VEGF gene silencing efficiency of A: phosphate buffered saline (PBS), B: Thiol-VEGF siRNA only, C: TP(3)-DA-PEI/thiol-scramble siRNA, D: bPEI25k/thiol-VEGF siRNA, E: DA-PEI/thiol-VEGF siRNA, and F: TP(3)-DA-PEI/thiol-VEGF siRNA polyplexes in A549 cells performed by ELISA assay. The results are presented as means ± SD and analyzed using Student’s *t*-test (*n* = 3, * *p* < 0.05).

The transfection and gene silencing ability of TP(3)-DA-PEI/thiol-siRNA were further evaluated using a therapeutic siRNA targeting vascular endothelial growth factor (VEGF). Blockage of neovascularization in solid tumor by the inhibition of VEGF expression has been considered as a promising therapeutic approach because VEGF is one of the crucial regulator of the tumor-induced angiogenesis [26,27]. Figure 5a,b showed the change of VEGF mRNA level in A549 cells after the transfection of the indicated formulations. The amount of VEGF protein after the transfection also showed a good correlation with the mRNA levels (Figure 5c). Among the formulations, TP(3)-DA-PEI/thiol-VEGF siRNA demonstrated superior suppression of VEGF expression, while TP(3)-DA-PEI complexed with thiol-siRNA with a scrambled sequence (TP(3)-DA-PEI/thiol-scRNA) failed for the suppression of the target gene, suggesting sequence-specific inhibition of target gene expression. In addition, the cytotoxicity of TP(3)-DA-PEI/thiol-siRNA at the transfection condition (polymer/siRNA weight ratio of 3) was negligible in A549 cells, as determined by measuring mitochondrial dehydrogenase activity using MTT assay (Figure S2).

### 3.4. Intracellular Uptake of TP-DA-PEI/thiol-siRNA

**Figure 6:**
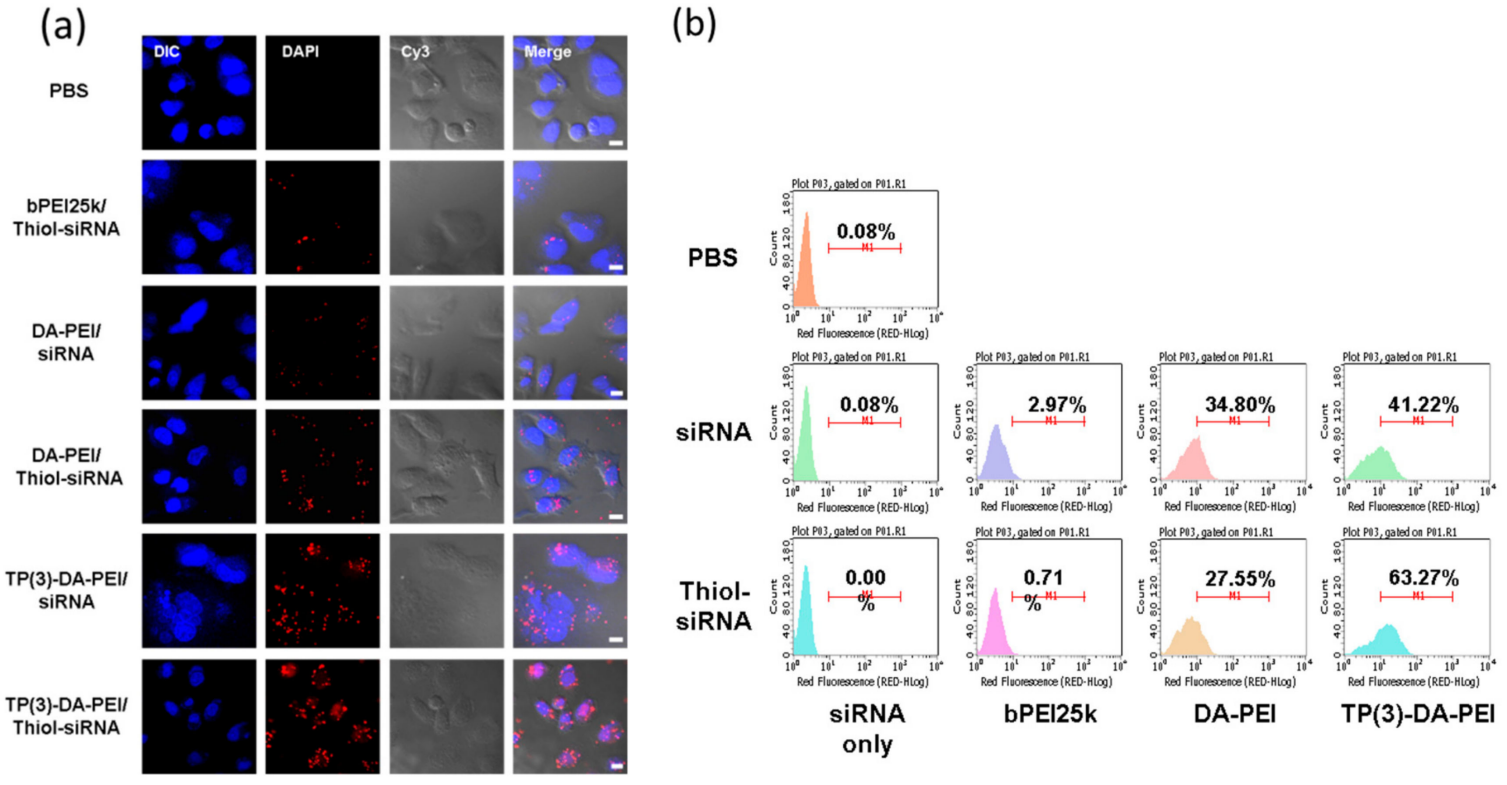
Intracellular uptake of TP(3)-DA-PEI/thiol-siRNA monitored by confocal microscope (**a**) and flow cytometry (**b**). A549 cells were treated with Cy3 labeled siRNA (or thiol-siRNA) complexed with DA-PEI or TP(3)-DA-PEI at polymer/siRNA weight ratio of 3, * *p* < 0.05. Polyplexes from bPEI25k and siRNA (or thiol-siRNA) (weight ratio = 1) were used as control. Nuclei were stained with DAPI (blue). Scale bars: 10 µm. M1 represents arbitrarily gated region.

The cellular uptake of TP-DA-PEI/thiol-siRNA by A549 cells was observed using confocal microscopy and flow cytometry using Cy3-labeled siRNA and thiol-siRNA. TP(3)-DA-PEI/thiol-siRNA demonstrated superior performance of cellular delivery of siRNA among the formulations (Figure 6). The enhanced cellular delivery efficiency of polyplexes with tight and compact structures using disulfide linkages can also be found in previous observations [28,29]. The limited stability of polyplex because of the short and rigid structure of siRNA could be improved by the formation of the disulfide linkages between thiol-siRNA and TP(3)-DA-PEI. In addition to disulfide linkages, the π–π interactions between the aromatic pendent groups may also contribute to the increased stability of the polyplex, which consequently resulted in the enhanced transfection of TP(3)-DA-PEI/thiol-siRNA.

### 3.5. In Vivo Biodistribution and Anti-Tumor Effect of TP-DA-PEI/thiol-siRNA

**Figure 7:**
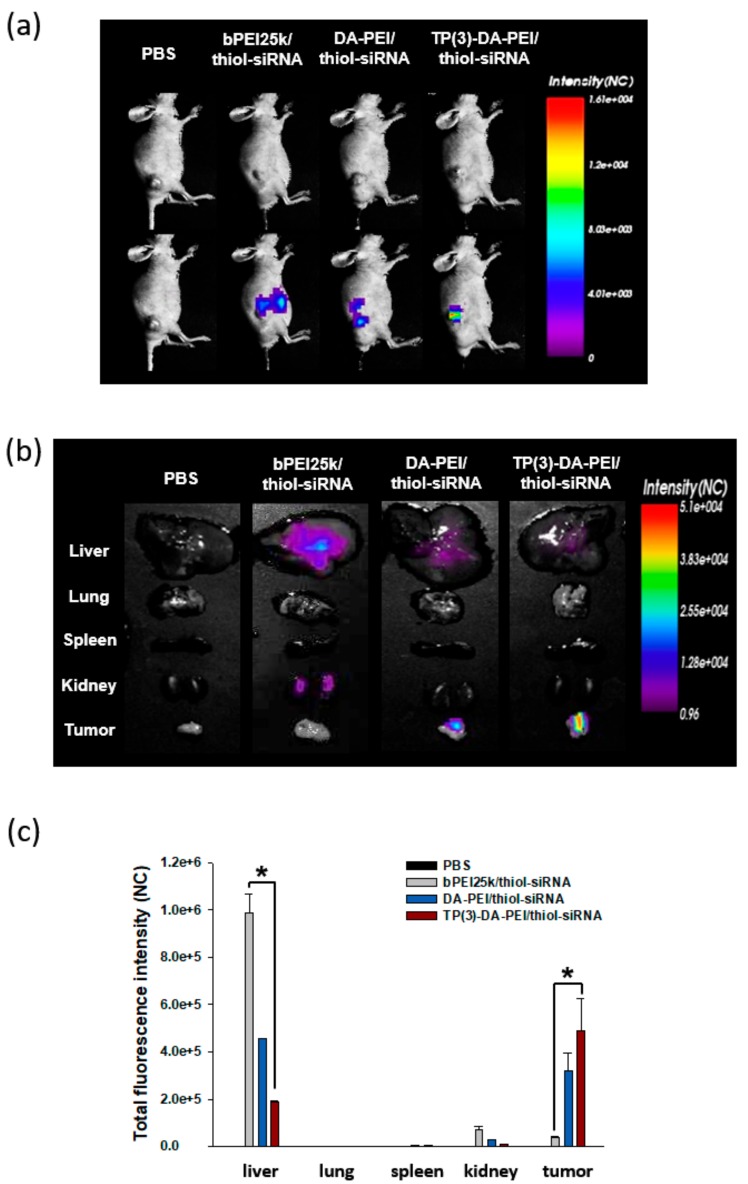
(**a**) In vivo imaging in A549 tumor of subcutaneous model-bearing nude mice at 12 h after intravenous injection of PBS, bPEI25k/Cy5.5-thiol-siRNA, DA-PEI/Cy5.5-thiol-siRNA, and TP(3)-DA-PEI/Cy5.5-thiol-siRNA polyplexes. (**b**) Representative images of dissected organs and tumors at 12 h after intravenous injection of these four polyplexes. (**c**) Corresponding total fluorescence intensity of organs and tumors. The results are presented as means ± SD and analyzed using Student’s *t*-test (*n* = 3, * *p* < 0.05).

To observe in vivo bio-distribution profiles of TP-DA-PEI/thiol-siRNA, Cy5.5 labeled thiol-siRNAs complexed with TP(3)-DA-PEI were intravenously administrated to A549 tumor bearing mice through the tail vein. Compared with bPEI25k/thiol-siRNA and DA-PEI/thiol-siRNA, TP(3)-DA-PEI/thiol-siRNA showed highest accumulation in the solid tumor 12 h after the systemic administration of the polyplexes (Figure 7). Interestingly, TP(3)-DA-PEI/thiol-siRNA exhibited relatively lower accumulation, especially in the liver and kidneys, compared with bPEI25k/thiol-siRNA and DA-PEI/thiol-siRNA. This may be because of the instability of bPEI25k/thiol-siRNA and DA-PEI/thiol-siRNA in the presence of serum proteins as shown in Figure 4. The polyplexes may be readily dissociated to release the siRNA during the systemic circulation, leading to degradation of siRNA. The resulting RNA oligomers with the fluorescent probe (Cy5.5) may be accumulated in the liver and filtered through the glomerular units of the kidneys, showing different biodistribution profiles from DA-PEI/thiol-siRNA, which provided secure protection of siRNA from degradation in 50% serum for 48 h. The predominant protective effect may result in the stable circulation in the blood stream. The stabilized polyplexes of TP(3)-DA-PEI/thiol-siRNA could be accumulated to the solid tumor as a result of the enhanced permeation and retention (EPR) effect [30].

**Figure 8:**
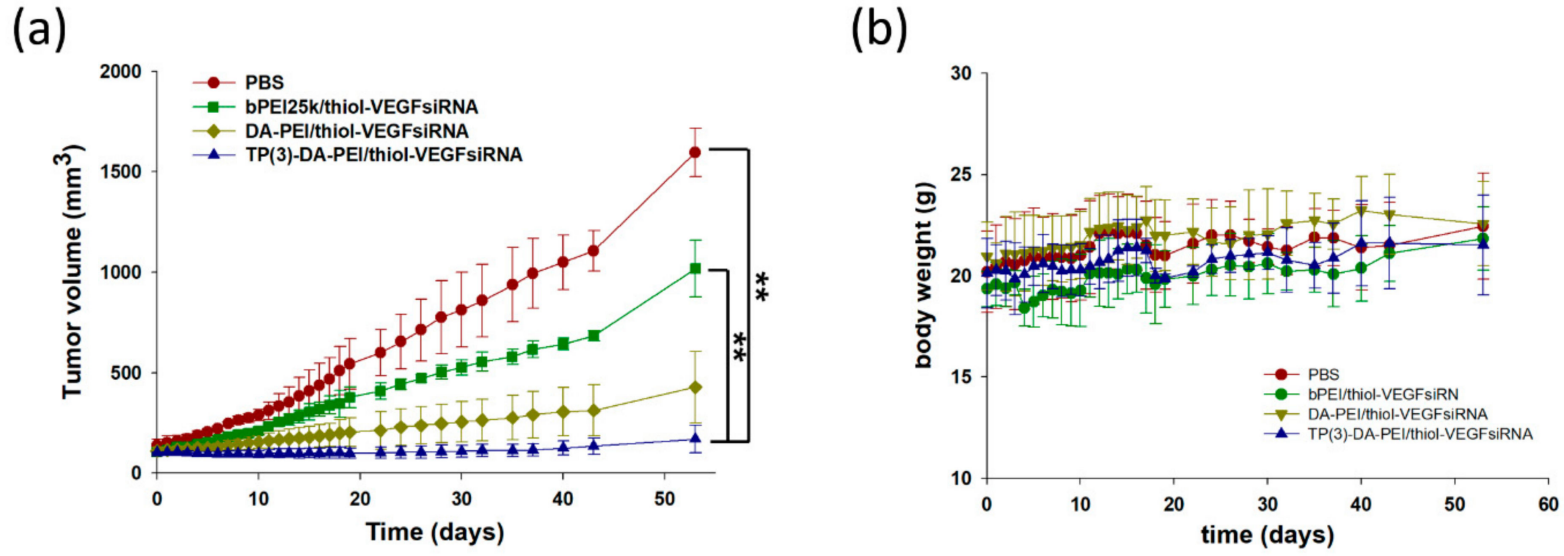
(**a**) Effect of tumor growth inhibition and (**b**) body weights of PBS, bPEI25k/thiol-VEGF siRNA, DA-PEI/thiol-VEGF siRNA, and TP(3)-DA-PEI/thiol-VEGF siRNA groups in A549 tumor-bearing mice via intravenous injection. The results are presented as means ± SD and analyzed using Student’s *t*-test (*n* = 5, ** *p* < 0.01).

The anti-tumor effects of TP(3)-DA-PEI/thiol-siRNA targeting VEGF were investigated after intravenous injection to mouse bearing A549 tumor xenograft. According to the tumor growth profiles for 50 days, TP(3)-DA-PEI/thiol-VEGF siRNA exhibited significant suppression of tumor growth in comparison with bPEI25k/siRNA or DA-PEI/siRNA (Figure 8a). The systemic administration of the formulations did not lead to weight loss of the animals during the experimental period (Figure 8b).

## 4. Conclusions

In this study, a conjugate of deoxycholic acid and low molecular weight PEI (M_W_ 1800) was further modified by introducing thiophenol groups, which provide a chance to form stable polyplex with thiol-siRNA via the formation of electrostatic interactions, disulfide bonds, and establishing π–π interactions. The resulting polymer (TP-DA-PEI) was able to form tight complex with thiol-siRNA and efficiently protect the siRNA in the presence of serum proteins. The polyplex of TP(3)-DA-PEI/thiol-siRNA targeting VEGF showed the enhanced accumulation in the solid tumor and effective inhibition of tumor growth in an animal tumor xenograft model. Considering its superior stability in serum environment and ability to deliver siRNA to tumor tissue, TP-DA-PEI conjugate could be considered as a candidate carrier for systemic cancer therapy using siRNA therapeutics.

## Figures and Tables

**Table 1:** The nomenclatures and compositions of TP-DA-PEI conjugates.

Polymer	Molar Feed Ratio (DA-PEI/FTP)	Composition ^1^ (DA-PEI/FTP)
TP(1)-DA-PEI	1:1	1:0.81
TP(3)-DA-PEI	1:3	1:2.13
TP(5)-DA-PEI	1:5	1:3.14

^1^ Determined by ^1^H NMR.

## Supplementary Materials

The following are available online at http://www.mdpi.com/2073-4360/10/9/953/s1, Figure S1: ^1^H NMR spectrum of TP(3)-DA-PEI in DMSO, Figure S2: Cytotoxicity of polyplexes formed between siRNA (or thiol-siRNA) and bPEI25k, DA-PEI, or TP(3)-DA-PEI in A549 cells.
