# Supplementary material for: Inhibition of Tumor Growth via Systemic siRNA Delivery Using Reducible Bile Acid-Conjugated Polyethylenimine
Source: Polymers (Basel). 2018 Aug 27;10(9):953. doi: 10.3390/polym10090953 (PMC6403700; doi:10.3390/polym10090953)
Supplement: Supplementary file 1 [file polymers-10-00953-s001.pdf]

polymers

MDPI

Supplementary information

Inhibition of Tumor Growth via Systemic siRNA Delivery Using Reducible Bile Acid-Conjugated Polyethylenimine

Yue Yin, Jung Eun Lee, Nak Won Kim, Jong Han Lee, Su Yeon Lim, E Seul Kim, Ji Won Park, Min Sang Lee * and Ji Hoon Jeong *

[1] School of Pharmacy, Sungkyunkwan University, Suwon 16419, Republic of Korea

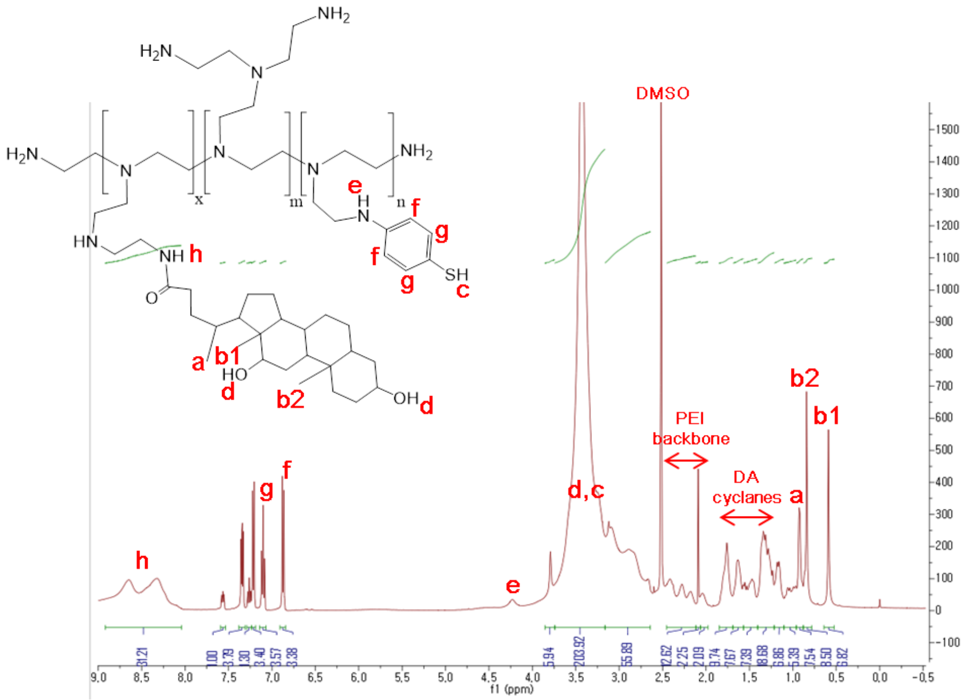

Figure S1. ^{1}H NMR spectrum of thiophenol (TP)(3)-deoxycholic acid (DA)-polyethylenimine (PEI) in dimethyl sulfoxide (DMSO).

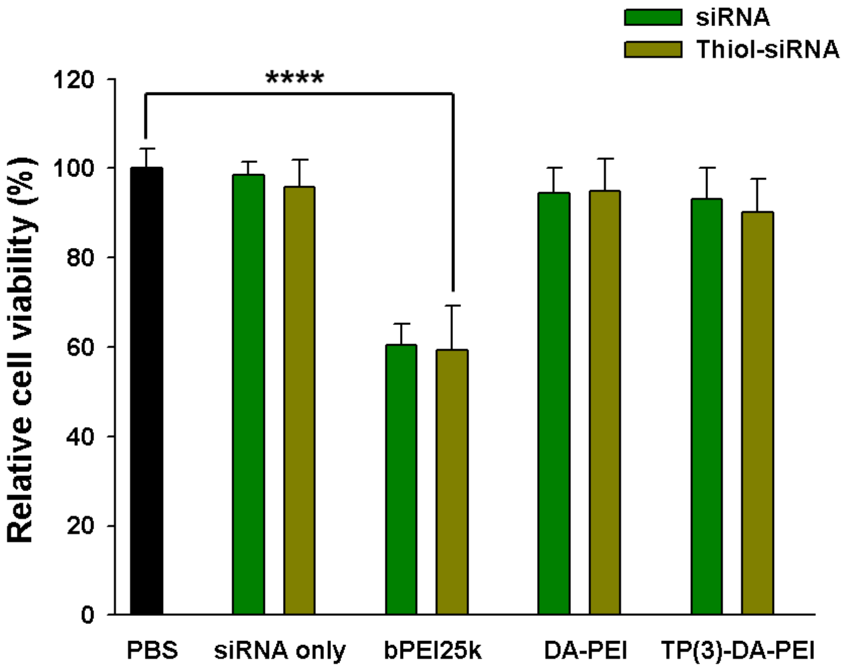

Figure S2. Cytotoxicity of polyplexes formed between small interfering RNA (siRNA) (or thiol-siRNA) and bPEI25k (polymer/siRNA weight ratio = 1), DA-PEI (weight ratio = 3), or TP(3)-DA-PEI (weight ratio = 3) in A549 cells. The fraction of viable cells was measured using 3-(4,5-dimethyl-2-thiazolyl)-2,5-diphenyl-2H-tetrazolium bromide (MTT) assay. The results are presented as means ± SD and analyzed using Student's t-test (n = 6, **** $p < 0.0001$).
